# Behavioral aspects and learning motivation: a study of middle school adolescents

**DOI:** 10.1590/2317-1782/20212021119

**Published:** 2022-04-08

**Authors:** Queila Pereira Antunes, Graziela Nunes Alfenas Fernandes, Stela Maris Aguiar Lemos

**Affiliations:** 1 Departamento de Fonoaudiologia, Faculdade de Medicina, Universidade Federal de Minas Gerais – UFMG - Belo Horizonte (MG), Brasil.

**Keywords:** Speech, Language and Hearing Sciences, Learning, Behavior, Motivation, Adolescence

## Abstract

**Purpose:**

To analyze the association between behavioral aspects and learning motivation according to age, sex, and grade in school in middle school students.

**Methods:**

Observational, analytical, and cross-sectional study with 11- to 14-year-old adolescents, who answered the participant characterization questionnaire, the Strengths and Difficulties Questionnaire – SDQ-Por, and the Learning Motivation Evaluation Scale – EMAPRE. Descriptive and bivariate statistical analyses were conducted.

**Results:**

In the sample researched, there was a statistically significant association between the Strengths and Difficulties Questionnaire domains and the learning motivation goals. It demonstrated that the students with higher means and medians for higher quality motivations had normal results in the SDQ conduct problems, whereas those with a greater tendency to a more extrinsic motivation had an abnormal result in peer relationship problems. In the total classification, the sample students with higher mean and median for the learning goal (which refers to a greater academic commitment) had a normal result, whereas those more prone to the performance-avoidance goal had more abnormal results. The learning motivation did not vary according to age and grade in school, and the adolescents had a greater tendency to the learning goal than to the other two.

**Conclusion:**

The association between the behavioral aspects and the learning motivation in the sample assessed was present in the abnormal SDQ-Por scores in relation to the performance-avoidance goal, and in the normal SDQ-Por scores in relation to the learning goal.

## INTRODUCTION

In academic life, from the first years of basic education to the beginning of higher education, the students are constantly exposed to scientific content that is unrelated to their experience and reality. Hence, academic commitment requires effort, intact brain functions, and a social context that help students achieve good results and carry on the learning process^(1)^.

Another point that stands out in this context is their motivation – i.e., the reason why students engage in tasks that have been proposed to them^(2)^. Studies point to learning motivation as one of the essential factors to favor learning^(3)^. Other researchers related the learning motivation to the students’ performance^(4)^.

Since motivation is a multidimensional and complex construct, some theories try to analyze it from different perspectives. The achievement goal theory, on which the presents study is based, outlines different types of motivation according to the “reasons for engagement”^(2)^ and encompasses the person’s manner of thinking, self-view, objectives, and emotions. These issues greatly influence their reaction to academic tasks^(5)^. Thus, motivation is classified into the “learning goal”, which refers to the student’s willingness to face the challenges inherent to learning and intellectual growth and their persistence in academic activities; the “performance-approach goal”, in which the student strives to show themselves competitive and stand out from the others; and the “performance-avoidance goal”, which refers to avoiding mistakes so they will not appear to be incapable^(6)^.

Therefore, it is understandable that factors extrinsic to the person, such as the family environment and socioeconomic conditions, influence their development at school, possibly leading to school difficulties^(7)^. Other factors are intrinsic to the person^(1)^, such as functional disabilities, hearing loss, and behavioral problems. All these difficulties require greater attention on their part to diminish the suffering that results from school failure and its consequences^(8)^.

A literature review pointed out the prevalence of learning difficulty associated with behavioral and emotional problems and other disorders, such as attention deficit, hyperactivity, and depression^(9)^. The research demonstrates that externalizing behaviors and poor academic skills are closely associated. Possible deviant behaviors can be identified as early as kindergarten, signaling later school difficulties^(9)^. Since learning difficulties are generally related to other comorbidities^(10)^, the underlying difficulties can supposedly be found from what has been manifested – the behavior.

The literature shows a relationship between poor school performance and clinically relevant emotional and/or behavioral symptoms^(11)^. In general, the student’s externalizing behaviors exhibited at school are characterized as oppositional, aggressive, hyperactive, impulsive, defiant, and antisocial. On the other hand, some students have internalizing behaviors, manifested as dysphoria (depression), withdrawal, fear, and anxiety^(9)^.

Hence, this study seeks to investigate students’ behavioral complaints. According to the literature^(10)^, behavioral issues are mostly noticed by the parents and point to a causal factor of great interest to the speech-language-hearing sciences – the learning difficulties. In-depth knowledge of the behavioral factors associated with learning complaints helps reach a differential diagnosis of the school difficulties and learning disorders^(1)^. It also helps prevent problems that originated in feelings related to the school environment, peer comparison, and the resulting feelings of inferiority, low self-esteem^(11)^, and so forth. Moreover, since they are adolescents, it helps understand the factors that lead middle school students to be more engaged with school tasks and more committed to academic activities. This is highly important for teachers to identify better strategies to motivate them and make the school setting more pleasant to them^(12)^.

The objective of this study was to analyze the association between behavioral aspects and learning motivation according to age, sex, and grade in school in middle schoolers. Specifically, the objectives of the paper were to describe the students’ behavioral capacities and difficulties and learning motivation, according to the domains: learning goal, performance-approach goal, and performance-avoidance goal, and verify the association of behavioral capacities and difficulties with learning motivation and their sex, age, and grade in school.

## METHODS

This study was approved by the Research Ethics Committee under evaluation report no. 2.422.795. It has an observational, analytical, cross-sectional design, with a nonprobabilistic sample comprising 124 adolescents 11 to 14 years old, who attended middle school at a private institution.

The inclusion criteria were as follows: 11- to 14-year-old students enrolled in middle school; who signed the informed assent form and whose parents/guardians signed the informed consent form; and who agreed to answer the questionnaires used in the study. Adolescents were excluded if they did not understand the instruments or had cognitive, neurological, or psychiatric impairments that prevented them from participating in the research.

The data were collected from the questionnaires filled in by the students. They were both allowed not to answer any questions that made them feel uncomfortable and assured that the information would remain secret and be used only in this research.

The instruments used in the study were the Strength and Difficulties Questionnaire (SDQ-Por)^(13)^, which has been used internationally and validated in Brazilian Portuguese, the Learning Motivation Assessment Scale (EMAPRE, its acronym in Portuguese)^(3)^, and a participant characterization questionnaire.

The SDQ has 25 items, classified into five scales, involving emotional symptoms (five items); conduct problems (five items); hyperactivity/inattention (five items); peer relationship problems (five items), and prosocial behavior (five items). It was filled in by the students because the sample age range began at 11 years. The protocol defines the total difficulty score above 20 points as “abnormal” and the prosocial behavior score up to 4 points as “abnormal”.

The EMAPRE, which assesses the learning motivation based on the achievement goal theory, is divided into three domains accepted by Brazilian researchers^(7)^ – the learning goal (12 items), the performance-approach goal (nine items), and the performance-avoidance goal (seven items) –, totaling 28 items in which the students chose from the following answer options: “I agree”, “I don’t know”, and “I disagree”.

Two response variables were used, namely: the students’ capacities (SDQ – prosocial behavior) and difficulties (SDQ – total), based on the SDQ. The explanatory variables were the learning motivation, sex, age, and grade in school.

Descriptive and bivariate statistical analyses were conducted with the frequency distribution of the categorical variables and the measures of central tendency and dispersion of the continuous variables. The association analyses were made with Pearson’s chi-squared, Kruskal-Wallis, and Mann-Whitney tests, with the statistical significance level set at p ≤ 0.05. The data were entered, processed, and analyzed in the SPSS software, version 25.0.

## RESULTS

Most participants in the sample were females (54.0%), 11 years old (27.4%), in sixth grade (32.2%). As for motivation, the learning goal had a higher mean and median than the performance-approach and performance-avoidance goals (Table 1).

**Table 1:** Descriptive analysis of age and the topics in the Learning Motivation Scale

**Variables**	**N**	**Mean**	**S.D.**	**Median**	**Minimum**	**Q_1_**	**Q_3_**	**Maximum**
Age (years)	124	12.44	1.12	12.00	11.00	11.00	13.00	14.00
Learning goal (sum)	124	29.28	4.65	31.00	16.00	26.00	32.75	36.00
Performance-approach goal (sum)	124	15.30	4.36	15.00	9.00	12.00	19.00	27.00
Performance-avoidance goal (sum)	124	9.40	3.10	8.00	7.00	7.00	10.00	21.00

**Caption:** N = number of participants; SD = standard deviation; Q = quartile

**Figure 1:**
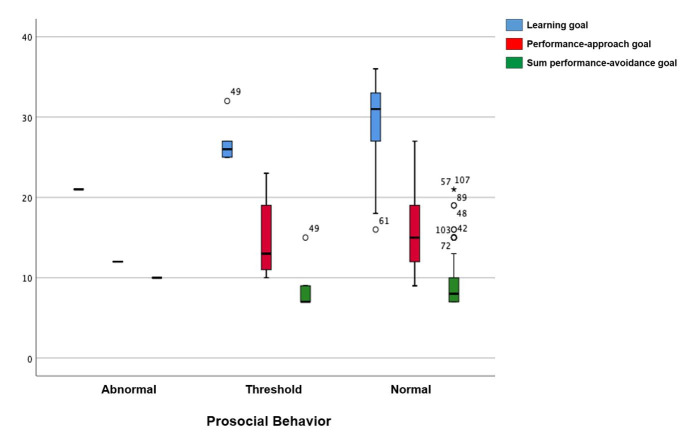
Boxplot of the prosocial behavior in the Strengths and Difficulties Questionnaire and the topics in the Learning Motivation Scale ‘o’ and ‘*’: outliers

**Figure 2:**
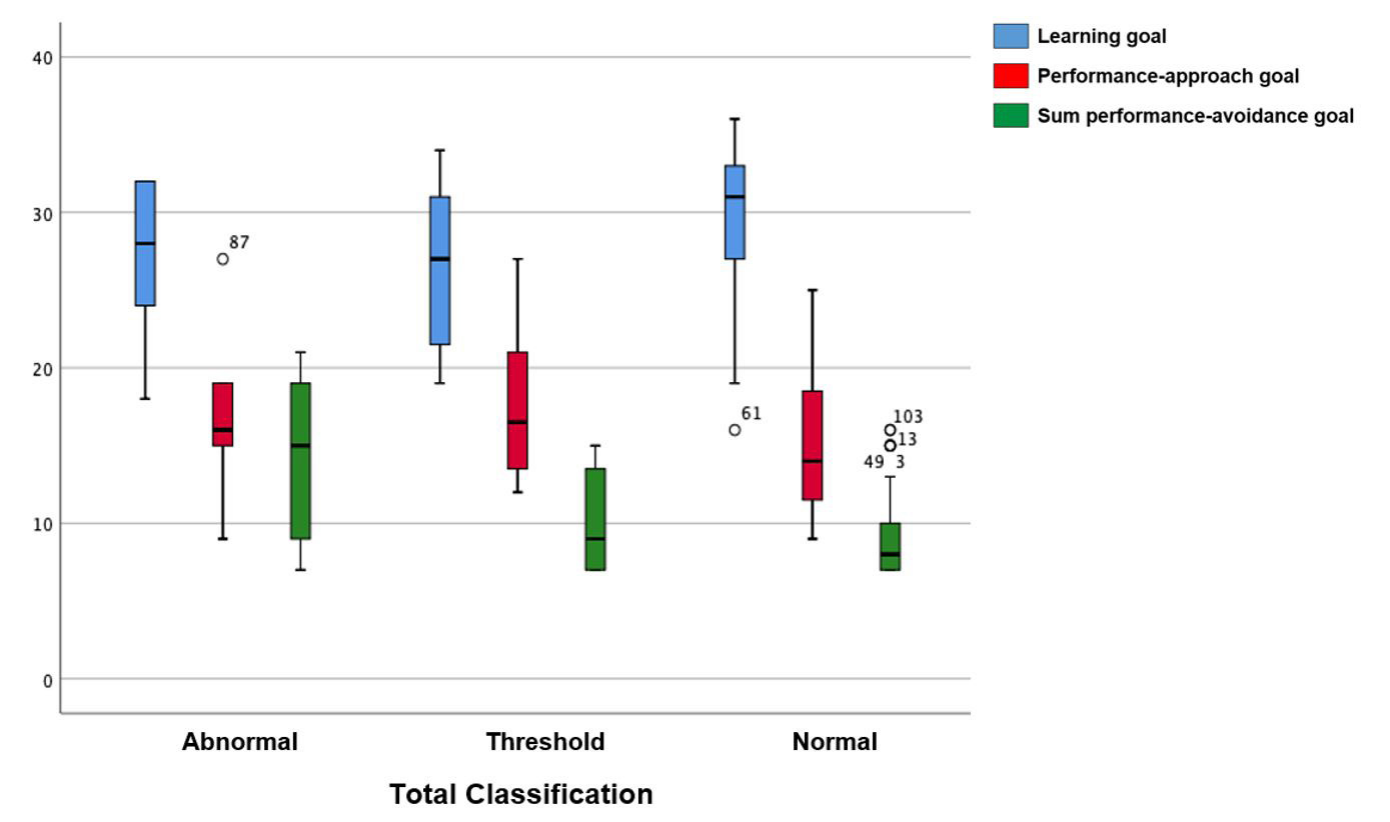
Boxplot of the total classification in the Strengths and Difficulties Questionnaire and the topics in the Learning Motivation Scale ‘o’: outliers

In the SDQ classification analysis (per scales, prosocial behavior, and total classification), most students had a normal result in all scales (Figures 1 and 2) – 67.7% in emotional symptoms; 72.6% in conduct problems; 71.8% in hyperactivity; and 75.8% in peer relationship problems. The normal results in prosocial behavior totaled 95.2%, and in the total score, 83,1%.

The association analysis of the SDQ prosocial behavior and total score with the numerical variables sex, age, and grade in school did not find statistically significant results. However, the data indicate that the emotional symptoms and peer relationship problems were more present in females than males, according to the SDQ. Of the adolescents whose scores pointed to abnormal behavior, 66.7% were girls and 33.3%, boys in the emotional symptoms; and, in the peer relationship problems, 61.5% were girls (Table 2).

**Table 2:** Association between the Strengths and Difficulties Questionnaire and the sociodemographic data

**SDQ**	**Sex**			**Age (years)**					**Grade in School**				
	**Fem. N (%)**	**Males N (%)**	**Total N (%)**	**11 N (%)**	**12 N (%)**	**13 N (%)**	**14 N (%)**	**Total N (%)**	**6^th^ N (%)**	**7^th^ N (%)**	**8^th^ N (%)**	**9^th^ N (%)**	**Total N (%)**
**Emotional Symptoms**													
Abnormal	16 (66.7)	8 (33.3)	24 (100.0)	4 (16.7)	7 (29.2)	8 (33.3)	5 (20.8)	24 (100.0)	5 (20.8)	9 (37.6)	5 (20.8)	5 (20.8)	24 (100.0)
Threshold	11 (68.8)	5 (31.2)	16 (100.0)	3 (18.8)	2 (12.5)	5 (31.2)	6 (37.5)	16 (100.0)	3 (18.8)	3 (18.8)	6 (37.5)	4 (25.1)	16 (100.0)
Normal	40 (47.6)	44 (53.4)	84 (100.0)	27 (32.1)	21 (25.0)	19 (22.6)	17 (20.2)	84 (100.0)	32 (38.1)	22 (26.1)	15 (17.9)	15 (17.9)	84 (100.0)
Total	67 (54.0)	57 (46.0)	124 (100.0)	34 (27.4)	30 (24.2)	32 (25.8)	28 (22.6)	124 (100.0)	40 (32.3)	34 (27.4)	26 (21.0)	24 (19.4)	124 (100.0)
p-value	0.115			0.405					0.321				
**Conduct Problems**													
Abnormal	10 (55.6)	8 (44.4)	18 (100.0)	6 (33.3)	5 (27.8)	5 (27.8)	2 (11.1)	18 (100.0)	6 (33.3)	6 (33.3)	4 (22.2)	2 (11.2)	18 (100.0)
Threshold	8 (50.0)	8 (50.0)	16 (100.0)	3 (18.8)	4 (25.0)	4 (25.0)	5 (31.2)	16 (100.0)	3 (18.8)	6 (37.5)	2 (12.5)	5 (31.2)	16 (100.0)
Normal	49 (54.4)	41 (45.6)	90 (100.0)	25 (27.8)	21 (23.3)	23 (25.6)	21 (23.3)	90 (100.0)	31 (34.4)	22 (24.4)	20 (22.3)	17 (18.9)	90 (100.0)
Total	67 (54.0)	57 (46.0)	124 (100.0)	34 (27.4)	30 (24.2)	32 (25.8)	28 (22.6)	124 (100.0)	40 (32.3)	34 (27.4)	26 (21.0)	24 (19.4)	124 (100.0)
p-value	0.938			0.875					0.596				
**Hyperactivity**													
Abnormal	8 (44.4)	10 (55.6)	18 (100.0)	2 (11.1)	3 (16.7)	8 (44.4)	5 (27.8)	18 (100.0)	2 (11.1)	5 (27.8)	8 (44.4)	3 (16.7)	18 (100.0)
Threshold	6 (35.3)	11 (64.7)	17 (100.0)	5 (29.4)	7 (41.2)	3 (17.6)	2 (11.8)	17 (100.0)	6 (35.3)	6 (35.3)	3 (17.6)	2 (11.8)	17 (100.0)
Normal	53 (59.6)	36 (40.4)	89 (100.0)	27 (30.3)	20 (22.5)	21 (23.6)	21 (23.6)	89 (100.0)	32 (36.0)	23 (25.8)	15 (16.9)	19 (21.3)	89 (100.0)
Total	67 (54.0)	57 (46.0)	124 (100.0)	34 (27.4)	30 (24.2)	32 (25.8)	28 (22.6)	124 (100.0)	40 (32.3)	34 (27.4)	26 (21.0)	24 (19.4)	124 (100.0)
p-value	0.125			0.187					0.139				
**Peer Relationship Problems**													
Abnormal	8 (61.5)	5 (38.5)	13 (100.0)	2 (15.4)	4 (30.7)	5 (38.5)	2 (15.4)	13 (100.0)	3 (23.0)	4 (30.8)	4 (30.8)	2 (15.4)	13 (100.0)
Threshold	10 (58.8)	7 (41.2)	17 (100.0)	8 (47.1)	1 (5.9)	6 (35.3)	2 (11.8)	17 (100.0)	8 (47.1)	3 (17.6)	4 (23.5)	2 (11.8)	17 (100.0)
Normal	49 (52.1)	45 (47.9)	94 (100.0)	24 (25.5)	25 (26.6)	21 (22.4)	24 (25.5)	94 (100.0)	29 (30.9)	27 (28.7)	18 (19.1)	20 (21.3)	94 (100.0)
Total	67 (54.0)	57 (46.0)	124 (100.0)	34 (27.4)	30 (24.2)	32 (25.8)	28 (22.6)	124 (100.0)	40 (32.3)	34 (27.4)	26 (21.0)	24 (19.4)	124 (100.0)
p-value	0.745			0.153					0.698				
**Prosocial Behavior**													
Abnormal	0 (0.0)	1 (100.0)	1 (100.0)	0 (0.0)	0 (0.0)	1 (100.0)	0 (0.0)	1 (100.0)	0 (0.0)	0 (0.0)	1 (100.0)	0 (0.0)	1 (100.0)
Threshold	2 (40.0)	3 (60.0)	5 (100.0)	0 (0.0)	4 (80.0)	0 (0.0)	1 (20.0)	5 (100.0)	1 (20.0)	3 (60.0)	0 (0.0)	1 (20.0)	5 (100.0)
Normal	65 (55.1)	53 (44.9)	118 (100.0)	34 (28.8)	26 (22.0)	31 (26.3)	27 (22.9)	118 (100.0)	39 (33.1)	31 (26.3)	25 (21.1)	23 (19.5)	118 (100.0)
Total	67 (54.0)	57 (46.0)	124 (100.0)	34 (27.4)	30 (24.2)	32 (25.8)	28 (22.6)	124 (100.0)	40 (32.3)	34 (27.4)	26 (21.0)	24 (19.4)	124 (100.0)
p-value	0.444			0.060					0.314				
**Total Classification**													
Abnormal	7 (77.8)	2 (22.2)	9 (100.0)	2 (22.2)	3 (33.3)	3 (33.3)	1 (11.2)	9 (100.0)	3 (33.3)	3 (33.3)	2 (22.2)	1 (11.2)	9 (100.0)
Threshold	6 (50.0)	6 (50.0)	12 (100.0)	3 (25.0)	3 (25.0)	3 (25.0)	3 (25.0)	12 (100.0)	3 (25.0)	3 (25.0)	3 (25.0)	3 (25.0)	12 (100.0)
Normal	54 (52.4)	49 (47.6)	103 (100.0)	29 (28.2)	24 (23.3)	26 (25.2)	24 (23.3)	103 (100.0)	34 (33.0)	28 (27.2)	21 (20.4)	20 (19.4)	103 (100.0)
Total	67 (54.0)	57 (46.0)	124 (100.0)	34 (27.4)	30 (24.2)	32 (25.8)	28 (22.6)	124 (100.0)	40 (32.3)	34 (27.4)	26 (21.0)	24 (19.4)	124 (100.0)
p-value	0.328			0.973					0.986				

Pearson’s chi-square test

**Caption:** N = number of participants; Fem. = females; SDQ = Strengths and Difficulties Questionnaire

The association analysis between the SDQ scales, prosocial behavior, and total score and the EMAPRE revealed statistically significant results between conduct problems and the learning goal (p=0.013), with a higher mean and median for the normal result – i.e., most students with the learning goal had a normal result in conduct problems. There was also an association between peer relationship problems and the performance-avoidance goal (p=0.002), with a higher mean for the abnormal result – i.e., in this sample, this goal is related to having more peer relationship problems. A statistically significant association was observed between the SDQ total classification and the learning goal (p=0.025), with a higher mean and median for the normal result; and with the performance-avoidance goal (p=0.012), with a higher mean and median for the abnormal result. There was an evident proportional relationship between the students’ goal preferences and externalizing behaviors (Table 3). Furthermore, the sample behavior was rather coherent in the comparison between the SDQ and EMAPRE, since the better the performance in prosocial behavior, the greater the preference for the learning goal. The analysis of the performance-avoidance goal revealed fewer adolescents with a normal SDQ result. It also revealed outlier points, whose values did not follow the normal sample pattern in the performance-avoidance goal. This suggests that positive behaviors are less frequent in students who approach learning with this goal (Figures 1 and 2).

**Table 3:** Analysis of the association between the Strengths and Difficulties Questionnaire and the topics in the Learning Motivation Scale

**Variables**	**Learning Goal**							**Performance-Approach Goal**						**Performance-Avoidance Goal**						
	**N**		**Mean**		**Median**		**S.D.**	**N**	**Mean**	**Median**		**S.D.**		**N**	**Mean**		**Median**		**S.D.**	
**Emotional Symptoms**																				
Abnormal	24		28.29		29.50		5.15	24	15.63	15.00		4.65		24	11.63		10.00		4.86	
Threshold	16		28.00		29.00		5.23	16	15.19	14.50		5.24		16	9.19		8.00		2.43	
Normal	84		29.81		31.00		4.35	84	15.23	14.50		4.14		84	8.80		8.00		2.19	
p-value	0.322							0.881						0.777						
**Conduct Problems**																				
Abnormal	18		26.06		26.00		5.49	18	17.22	16.50		4.72		18	10.44		8.50		4.29	
Threshold	16		28.19		28.50		5.13	16	15.31	14.50		4.57		16	9.00		7.50		3.41	
Normal	90		30.12		31.00		4.08	90	14.91	13.50		4.19		90	9.26		9.00		2.74	
p-value	0.013*							0.168						0.360						
**Hyperactivity**																				
Abnormal	18		27.22		27.50		5.50	18	16.83	16.50		4.72		18	11.11		8.50		4.91	
Threshold	17		28.12		29.00		3.60	17	14.94	14.00		4.42		17	10.24		9.00		3.90	
Normal	89		29.92		31.00		4.50	89	15.06	15.00		4.26		89	8.89		8.00		2.23	
p-value	0.059							0.300						0.261						
**Peer Relationship Problems**																				
Abnormal	13		27.69		28.00		5.19	13	16.54	16.00		5.41		13	12.08		10.00		4.41	
Threshold	17		28.94		31.00		6.37	17	15.76	15.00		4.66		17	9.88		10.00		2.40	
Normal	94		29.56		31.00		4.21	94	15.04	14.50		4.16		94	8.94		8.00		2.92	
p-value	0.470							0.622						0.002*						
**Prosocial Behavior**																				
Abnormal	1	21.00		21.00		0.00		1	12.00		12.00		0.00	1		10.00		10.00		0.00
Threshold	5	27.00		26.00		2.92		5	8.26		13.00		5.59	5		9.00		7.00		3.46
Normal	118	29.45		31.00		4.65		118	15.33		15.00		4.34	118		9.41		8.00		3.11
p-value	0.105							0.715						0.585						
**Total Classification**																				
Abnormal	9	26.56		28.00		5.55		9	16.33		16.00		5.50	9		14.33		15.00		5.85
Threshold	12	26.33		27.00		5.25		12	17.50		16.50		4.64	12		10.08		9.00		3.32
Normal	103	29.86		31.00		4.82		103	14.95		14.00		4.18	103		8.88		8.00		5.22
p-value	0.025*							0.151						0.012*						

Kruskal-Wallis test

* = p-value ≤ 0.05

**Caption:** N = number of participants; S.D. = standard deviation

## DISCUSSION

This study showed an association between the conduct problems and the learning goal. There were also relationships between peer relationship problems and the performance-avoidance goal; the learning goal and the SDQ total normal classification; and the performance-avoidance goal and SDQ total abnormal classification.

The greater tendency to the learning goal showed a greater inclination to an intrinsic motivation, which is associated in the literature with better academic performance and behavior less indicative of clinical anxiety. The teachers recognize such students as the ones that learn the most and have the highest academic self-knowledge and self-control indices in general^(14)^. Other studies point out that, as students develop greater self-effectiveness, they also acquire greater emotional management skills, which optimizes their learning^(15,16)^. Recent research mentions that healthy life habits greatly potentialize academic motivation^(16)^. Nevertheless, the relationship between motivation and academic performance is not a determinant, making it evident that the pedagogical practices, although traditionally based on extrinsic motivation, greatly influence the students’ willingness to do the school tasks^(14)^.

The greatest tendency to the learning goal was an expected result since the sample did not present school complaints. This corroborates the literature, as a more positive and lasting motivation shows that behavioral issues may not only result from possible school difficulties but also precede them, in many cases^(17)^. Another factor to consider has been observed in a study^(8)^ that points out how students internalize their teachers’ beliefs regarding their academic performance. According to Hareli and Weiner, “every student is sensitive to other people’s reactions, both the real and the potential ones, with consequences to their own judgments and behaviors “. Hence, they certify that the students’ self-perception is also influenced by the teachers’ feedback. In their turn, they respond to their teachers’ beliefs with their behavior, which may manifest as anger, withdrawal, or even greater exposure to the challenges, as seen in the attitude of those who mostly fit the learning goal. This demonstrates that this tendency is also shaped by the context to which each student belongs. A study conducted in a higher education setting observed that motivation is influenced by the environmental dynamics and the professors’ manner of carrying out academic activities. This demonstrates that the students’ greater freedom to ask their questions aloud, dialoguing with the groups and the professor, helped them be more successful in learning, leading them to be more involved in the tasks^(18)^. Thus, when the students’ autonomy is stimulated, along with other basic psychological needs, they also develop greater intrinsic motivation^(19)^.

Even though no statistically significant association was verified between the social behaviors and the sociodemographic data, the females’ greater tendency to have emotional symptoms and peer relationship problems is compatible with the data indicated in the literature. Studies on the behavior patterns between the sexes point to more frequent emotional symptoms (such as headache, stomachache, nausea, discouragement, crying spells, loss of confidence, and so on) in girls, whereas boys have more aggressive behaviors, with a greater tendency to hyperactivity^(20,21)^. This data must be further investigated in samples with more sociodemographic diversification.

The association between the SDQ and EMAPRE revealed that the adolescents that tended more to the learning goal had fewer conduct problems – corroborating the literature^(6)^, which reports that the learning goal is related to the intrinsic motivation – i.e., a positive behavior. This correlation is again made evident in the total classification of the association of the SDQ and EMAPRE, as more adolescents opted for the learning goal in the normal behavioral score. A piece of research^(22)^ compared students diagnosed with attention-deficit/hyperactivity disorder (ADHD) with children with typical development; it made evident the correlation between the disorder and the difficulties engaging in academic tasks. Hence, these students’ behavior is compatible with extrinsic motivation.

The behavioral changes occur along with the performance-avoidance goal in its association with peer relationship problems and SDQ total classification. This result is compatible with a study^(2)^ that observed a tendency to the performance-avoidance goal associated with low academic interest, anxiety, and poorer school performance. This also corroborates recent research^(23)^ conducted at the Center for Attention, Learning, and Memory of the Cambridge University, United Kingdom, with a mixed sample of students with school difficulties, either having associated disorders or not. The study made an association between two scales, the SDQ and the RCADS-P (the Revised Child Anxiety and Depression Scale), which assesses anxiety and depressive disorders in children. It verified that a large part of the sample had an abnormal behavior in the SDQ emotional symptoms (49%) and that the students with greater emotional symptoms and hyperactivity pointed to anxiety and depression problems. Another study^(24)^ investigated children and demonstrated a greater presence of behavioral comorbidities (with low self-esteem, greater social interaction difficulties, and aggressive behavior) in those with developmental disorders associated with school difficulties than in those with the diagnosis alone. These data highlight the importance of correlating, as in the present study, tests that encompass different factors involved in the learning process.

The sample homogeneity in terms of socioeconomic data and cultural aspects can be considered a limitation of this study. The students attended a private school and thus do not represent the vast majority of the Brazilian reality.

It must be emphasized that the advancements that have been attained help understand the associations between behavioral aspects and learning motivation, as few studies have such a triangulation, particularly using the EMAPRE and SDQ protocols and encompassing the age group presented here. Therefore, this evidence may contribute to the discussion on the topic on the part of health and education professionals who deal with students in their daily routine.

Given the above, the study on the topic point to the integration of learning with emotional aspects, based on a multifactorial perspective. This aims to highlight a subjective and greatly important aspect in the teaching and learning processes, namely, learning motivation. Hence, this study has a role in the dialogue between the knowledge of the various fields, contributing to the discussion and broadening the understanding of the processes related to the learning situations.

## CONCLUSION

This research demonstrated the association between behavioral aspects and learning motivation in middle school adolescents, with the association between the SDQ-Por abnormal scores and the performance-avoidance goal, and between the SDQ-Por normal scores and the learning goal. There was a statistically significant association between conduct problems and the learning goal; between peer relationship problems and the performance-avoidance goal; between the learning goal and the SDQ total normal classification; and between the performance-avoidance goal and the SDQ total abnormal classification. The investigated sample had no statistically significant difference in the behavioral aspects and learning motivation according to age, sex, and grade in school.

Therefore, understanding the relationship between behavioral aspects and learning motivation favors a multidisciplinary approach to adolescents, both in school and speech-language-hearing clinical practice, aiming at the well-being and better quality of life of the population studied.
