# Supplementary material for: Behavioral aspects and learning motivation: a study of middle school adolescents
Source: Codas. 2022 Apr 8;34(5):e20210119. doi: 10.1590/2317-1782/20212021119 (PMC9886179; doi:10.1590/2317-1782/20212021119)
Supplement: Translation

# Translation: Aspectos comportamentais e motivação para aprender: um estudo com adolescentes do ensino fundamental

**Authors:** Queila Pereira Antunes, Graziela Nunes Alfenas Fernandes, Stela Maris Aguiar Lemos

**Affiliations:** 1 Departamento de Fonoaudiologia, Faculdade de Medicina, Universidade Federal de Minas Gerais – UFMG - Belo Horizonte (MG), Brasil.

**Keywords:** Fonoaudiologia, Aprendizagem, Comportamento, Motivação, Adolescente

## Abstract

**Objetivo:**

analisar a associação entre aspectos comportamentais e motivação para aprender segundo idade, gênero e ano escolar em estudantes do ensino fundamental II.

**Método:**

estudo observacional, analítico e transversal com adolescentes de 11-14 anos, que responderam aos instrumentos de Questionário de caracterização dos participantes, Capacidades e Dificuldades – SDQ-Por, e Escala de Avaliação da Motivação para a Aprendizagem- EMAPRE. Foram realizadas análises estatísticas descritiva e bivariada.

**Resultados:**

na amostra pesquisada houve associação com significância estatística entre os domínios do instrumento de Capacidades e Dificuldades e metas relacionadas à motivação para aprender, que demonstrou que os estudantes que possuem maiores médias e medianas para uma motivação de melhor qualidade apresentaram resultados normais no domínio do SDQ “Problemas de Conduta”, enquanto que os que apresentaram maior tendência a uma motivação mais extrínseca, tiveram resultado anormal no domínio “Problemas no relacionamento com pares”. Na “Classificação total”, os estudantes da amostra com maior média e mediana para a Meta Aprender, que diz respeito a um maior empenho acadêmico, obtiveram resultado normal, já nos mais inclinados à Meta Performance Evitação, observou-se mais resultados anormais. A motivação para aprender não variou de acordo com os fatores de idade e ano escolar e os adolescentes apresentaram maior tendência à Meta Aprender se comparada às demais metas para aprendizagem.

**Conclusão:**

A associação entre os aspectos comportamentais e a motivação para aprender na amostra avaliada foi evidenciada entre os escores alterados do SDQ-Por e a Meta Performance Evitação e entre os escores normais do SDQ-Por e a Meta Aprender.

## INTRODUÇÃO

No percurso acadêmico, desde os anos iniciais da educação básica ao ingresso no ensino superior, ocorre constante exposição a conteúdos científicos que fogem da vivência e da realidade dos estudantes. De forma que o engajamento acadêmico exige esforço e funções cerebrais íntegras, bem como um contexto social favorável para a obtenção de bons resultados e a garantia da permanência do estudante no processo de aprendizado^(1)^.

Nesse contexto, deve ser destacada a motivação do indivíduo, ou seja, o porquê do estudante se engajar em alguma tarefa que lhe é proposta^(2)^. Estudos apontam a motivação para aprender como um dos fatores essenciais de favorecimento da aprendizagem^(3)^. Outros pesquisadores relacionaram a motivação para aprender ao desempenho do estudante^(4)^.

Sendo a motivação um construto considerado multidimensional e complexo, algumas teorias tentam analisá-la a partir de diferentes perspectivas. A Teoria da Meta de Realização, na qual se fundamenta o presente estudo, traça diferentes tipos de motivação a partir das “razões para o engajamento”^(2)^ e envolve a forma como o indivíduo pensa, a visão a respeito de si mesmo, os seus objetivos e emoções, questões que vão ter grande influência em como o indivíduo reage frente às tarefas acadêmicas^(5)^. Assim, a motivação é classificada em “meta aprender”, que diz respeito ao desejo do aluno em enfrentar os desafios inerentes à aprendizagem, ao crescimento intelectual, bem como à persistência nas atividades acadêmicas, “meta performance aproximação”, na qual o aluno busca parecer competente e se destacar em relação aos demais, e “meta performance evitação”, que diz respeito a evitar erros, para não parecer incapaz^(6)^.

Portanto, é compreensível que fatores extrínsecos ao indivíduo, como ambiente familiar e condições socioeconômicas, influenciem no desenvolvimento do estudante no ambiente escolar, e possam desencadear dificuldades escolares^(7)^. Outros fatores são intrínsecos ao sujeito^(1)^, como deficiências funcionais, perda auditiva, e problemas comportamentais. Todas essas dificuldades pressupõem maior atenção a estes indivíduos, para diminuir os sofrimentos decorrentes do fracasso escolar e suas consequências^(8)^.

Uma revisão de literatura apontou que há prevalência da dificuldade de aprendizagem atrelada a problemas comportamentais e emocionais, bem como a outros transtornos, como déficit de atenção, hiperatividade e depressão^(9)^. A pesquisa evidencia que os comportamentos externalizados e as habilidades acadêmicas pobres estão intimamente associados. Ainda na educação infantil é possível identificar de maneira precoce possíveis desvios de comportamento que sinalizam dificuldades escolares posteriores^(9)^ e uma vez que as dificuldades de aprendizado, em geral, estão atreladas a outras comorbidades^(10)^, entende-se ser possível encontrar as dificuldades de base, a partir do que é exposto, o comportamento.

A literatura apresenta relação entre o baixo desempenho escolar e sintomas emocionais e/ou comportamentais em níveis considerados de relevância clínica^(11)^. Em geral, os comportamentos externalizados pelos alunos em ambientes escolares se destacam como opositivos, agressivos, hiperativos, impulsivos, desafiadores e manifestação anti-social. Enquanto há quem apresente aspectos que ficam internalizados, que se evidenciam como: disforia (depressão), retraimento, medo e ansiedade^(9)^.

O presente estudo propõe, assim, investigações a respeito das queixas comportamentais dos escolares. De acordo com a literatura^(10)^, as questões comportamentais são mais percebidas pelos pais e apontam para um fator causal de grande interesse da fonoaudiologia, a dificuldade de aprendizagem. Aprofundar nos conhecimentos dos fatores comportamentais envolvidos com as queixas de aprendizagem permite realizar um diagnóstico diferencial quanto às dificuldades escolares e aos transtornos de aprendizagem^(1)^ e contribuir para a prevenção de problemas advindos dos sentimentos em relação ao ambiente escolar, das comparações com os pares e consequente sentimento de inferioridade, da baixa autoestima^(11)^, dentre outros. Além disso, uma vez que se trata do público adolescente, entender os fatores que contribuem para o engajamento nas propostas escolares e o comprometimento nas atividades acadêmicas é de fundamental importância para que o professor saiba identificar melhores estratégias de motivação a fim de tornar o ambiente escolar mais agradável^(12)^.

O presente estudo teve como objetivo analisar a associação entre os aspectos comportamentais e a motivação para aprender segundo idade, gênero e ano escolar em estudantes do Ensino Fundamental II. De maneira específica, os objetivos do trabalho foram descrever as capacidades e dificuldades comportamentais dos estudantes e a motivação para aprender segundo os domínios: meta aprender, meta performance-aproximação e meta performance-evitação e verificar a associação das capacidades e dificuldades comportamentais com a motivação para aprender e os seus domínios gênero, idade e ano escolar.

## MÉTODO

O presente estudo foi aprovado pelo Comitê de Ética em Pesquisa sob parecer de número 2.422.795 e apresenta delineamento observacional, analítico e transversal com amostra não probabilística composta por 124 adolescentes de 11 a 14 anos matriculados no ensino fundamental de uma escola de financiamento privado.

Os critérios de inclusão foram ser estudante matriculado no Ensino Fundamental II na faixa etária de 11 a 14 anos de idade, cujos responsáveis tenham assinado o Termo de Consentimento Livre e Esclarecido (TCLE), e os adolescentes que assinaram o Termo de Assentimento Livre e Esclarecido (TALE) e que aceitaram responder aos questionários propostos. Foram excluídos adolescentes que não compreenderam os instrumentos ou com alterações cognitivas, neurológicas ou psiquiátricas que impedissem a realização da pesquisa.

A coleta dos dados foi realizada por autopreenchimento dos estudantes, que foram informados que poderiam se recusar a responder alguma pergunta, caso se sentissem constrangidos, além de lhes ser assegurado o sigilo das informações para uso exclusivo da pesquisa em questão.

Os instrumentos utilizados para o estudo foram o Questionário de Capacidades e Dificuldades – SDQ-Por – *Strengths and Difficulties Questionnaire* – SDQ^(13)^, instrumento internacionalmente usado e validado para o português brasileiro, a Escala de Avaliação da Motivação para a Aprendizagem- EMAPRE^(3)^ e um questionário de caracterização dos participantes.

O SDQ é composto por 25 itens, classificados em cinco subcategorias que envolvem: sintomas emocionais (cinco itens); problemas de comportamento (cinco itens); hiperatividade/desatenção (cinco itens); problemas de relacionamento com colegas (cinco itens) e comportamento pró-social (cinco itens). O preenchimento foi realizado pelos próprios estudantes, uma vez que a amostra contou com a faixa etária acima de 11 anos de idade. O protocolo define o escore total de dificuldades acima de 20 pontos como “anormal” e o escore do “Comportamento pró-social” até 4 pontos como “anormal”.

A Escala de Avaliação da Motivação para a Aprendizagem – EMAPRE, que avalia a motivação para aprender baseado na Teoria de Metas de Realização, sendo distribuída em três domínios aceitos por pesquisadores brasileiros^(7)^: meta aprender (12 itens), meta performance-aproximação (9 itens) e meta performance-evitação (7 itens); totalizando 28 itens em que o aluno respondia de acordo com as opções “Concordo”, “Não sei” ou “Discordo”.

Foram elencadas duas variáveis respostas que foram as capacidades (SDQ-Comportamento pró-social) e dificuldades (SDQ-total) dos estudantes a partir do questionário SDQ. As variáveis explicativas foram a motivação para aprender, o gênero, a idade e o ano escolar.

Foram realizadas as análises estatísticas descritiva e bivariada com distribuição de frequência das variáveis categóricas e das medidas de tendência central e de dispersão das variáveis contínuas. Para as análises de associação foram utilizados os testes Qui-quadrado de Pearson, Kruskal-Wallis e Mann-Whitney, sendo considerados com significância estatística as que apresentaram valor de p ≤ 0,05. Para entrada, processamento e análise dos dados foi utilizado o software SPSS, versão 25.0.

## RESULTADOS

Na amostra pesquisada, a maioria dos participantes pertencia ao gênero feminino (54,0%), a maior parte possuía 11 anos de idade (27,4%) e cursava o 6o ano escolar (32,2%). Com relação às metas de motivação para aprendizagem, a Meta Aprender apresentou maior média e mediana se comparada às Metas Performance-Aproximação e Meta Performance-Evitação (Tabela 1).

**Tabela 1:** Análise descritiva das variáveis idade e dos tópicos da Escala de Motivação para Aprendizagem

**Variáveis**	**N**	**Média**	**D.P.**	**Mediana**	**Mínimo**	**1º Q**	**3º Q**	**Máximo**
Idade (anos)	124	12,44	1,12	12,00	11,00	11,00	13,00	14,00
Meta Aprender (soma)	124	29,28	4,65	31,00	16,00	26,00	32,75	36,00
Meta Performance Aproximação (soma)	124	15,30	4,36	15,00	9,00	12,00	19,00	27,00
Meta Performance Evitação (soma)	124	9,40	3,10	8,00	7,00	7,00	10,00	21,00

**Legenda:** N = número de participantes; DP = desvio padrão; Q = quartil

Na análise de classificação do questionário SDQ, por escalas, comportamento pró-social e classificação total foi possível verificar que em todas as escalas a maioria dos estudantes apresentou resultado considerado normal (Figuras 1 e 2), sendo 67,7% na Escala Sintomas Emocionais, 72,6% na Escala Problemas de Conduta, 71,8% na Escala Hiperatividade, e 75,8% na Escala de Problemas de Relacionamento com os colegas. O total de resultados normais no Comportamento Pró-Social foi de 95,2%; e no Escore total, 83,1% dos estudantes apresentaram-se dentro da normalidade.

**Figura 1:** Boxplot do Comportamento Pró-Social do SDQ e dos tópicos da Escala de Motivação para Aprendizagem ‘o’ e ‘*’ são valores discrepantes

**Figura 2:** Boxplot da Classificação Total do SDQ e dos tópicos da Escala de Motivação para Aprendizagem ‘o’ são valores discrepantes

Embora a análise de associação das escalas do comportamento pró-social e escore total do questionário SDQ com as variáveis numéricas “gênero”, “idade” e “ano escolar” não tenha demonstrado resultados com significância estatística, os dados indicaram que os sintomas emocionais e problemas de relacionamento com colegas tiveram maior expressão no gênero feminino, de acordo com o questionário SDQ, se comparado ao gênero masculino. Dentre os adolescentes que obtiveram pontuações que apontaram para alguma anormalidade comportamental, 66,7% eram meninas para 33,3% dos meninos, nos critérios de sintomas emocionais. Nos problemas de relacionamento com colegas, dentro do grupo que pontuou para anormalidade, 61,5% eram meninas (Tabela 2).

**Tabela 2:** Associação entre o questionário SDQ e dados demográficos

**SDQ**	**Gênero**			**Idade (anos)**					**Ano Escolar**				
	**Fem.**	**Masc.**	**Total**	**11**	**12**	**13**	**14**	**Total**	**6º**	**7º**	**8º**	**9º**	**Total**
	**N (%)**	**N (%)**	**N (%)**	**N (%)**	**N (%)**	**N (%)**	**N (%)**	**N (%)**	**N (%)**	**N (%)**	**N (%)**	**N (%)**	**N (%)**
**Sintomas Emocionais**													
Anormal	16 (66,7)	8 (33,3)	24 (100,0)	4 (16,7)	7 (29,2)	8 (33,3)	5 (20,8)	24 (100,0)	5 (20,8)	9 (37,6)	5 (20,8)	5 (20,8)	24 (100,0)
Limítrofe	11 (68,8)	5 (31,2)	16 (100,0)	3 (18,8)	2 (12,5)	5 (31,2)	6 (37,5)	16 (100,0)	3 (18,8)	3 (18,8)	6 (37,5)	4 (25,1)	16 (100,0)
Normal	40 (47,6)	44 (53,4)	84 (100,0)	27 (32,1)	21 (25,0)	19 (22,6)	17 (20,2)	84 (100,0)	32 (38,1)	22 (26,1)	15 (17,9)	15 (17,9)	84 (100,0)
Total	67 (54,0)	57 (46,0)	124 (100,0)	34 (27,4)	30 (24,2)	32 (25,8)	28 (22,6)	124 (100,0)	40 (32,3)	34 (27,4)	26 (21,0)	24 (19,4)	124 (100,0)
p-valor	0,115			0,405					0,321				
**Problemas de Conduta**													
Anormal	10 (55,6)	8 (44,4)	18 (100,0)	6 (33,3)	5 (27,8)	5 (27,8)	2 (11,1)	18 (100,0)	6 (33,3)	6 (33,3)	4 (22,2)	2 (11,2)	18 (100,0)
Limítrofe	8 (50,0)	8 (50,0)	16 (100,0)	3 (18,8)	4 (25,0)	4 (25,0)	5 (31,2)	16 (100,0)	3 (18,8)	6 (37,5)	2 (12,5)	5 (31,2)	16 (100,0)
Normal	49 (54,4)	41 (45,6)	90 (100,0)	25 (27,8)	21 (23,3)	23 (25,6)	21 (23,3)	90 (100,0)	31 (34,4)	22 (24,4)	20 (22,3)	17 (18,9)	90 (100,0)
Total	67 (54,0)	57 (46,0)	124 (100,0)	34 (27,4)	30 (24,2)	32 (25,8)	28 (22,6)	124 (100,0)	40 (32,3)	34 (27,4)	26 (21,0)	24 (19,4)	124 (100,0)
p-valor	0,938			0,875					0,596				
**Hiperatividade**													
Anormal	8 (44,4)	10 (55,6)	18 (100,0)	2 (11,1)	3 (16,7)	8 (44,4)	5 (27,8)	18 (100,0)	2 (11,1)	5 (27,8)	8 (44,4)	3 (16,7)	18 (100,0)
Limítrofe	6 (35,3)	11 (64,7)	17 (100,0)	5 (29,4)	7 (41,2)	3 (17,6)	2 (11,8)	17 (100,0)	6 (35,3)	6 (35,3)	3 (17,6)	2 (11,8)	17 (100,0)
Normal	53 (59,6)	36 (40,4)	89 (100,0)	27 (30,3)	20 (22,5)	21 (23,6)	21 (23,6)	89 (100,0)	32 (36,0)	23 (25,8)	15 (16,9)	19 (21,3)	89 (100,0)
Total	67 (54,0)	57 (46,0)	124 (100,0)	34 (27,4)	30 (24,2)	32 (25,8)	28 (22,6)	124 (100,0)	40 (32,3)	34 (27,4)	26 (21,0)	24 (19,4)	124 (100,0)
p-valor	0,125			0,187					0,139				
**Problemas de relacionamento com os colegas**													
Anormal	8 (61,5)	5 (38,5)	13 (100,0)	2 (15,4)	4 (30,7)	5 (38,5)	2 (15,4)	13 (100,0)	3 (23,0)	4 (30,8)	4 (30,8)	2 (15,4)	13 (100,0)
Limítrofe	10 (58,8)	7 (41,2)	17 (100,0)	8 (47,1)	1 (5,9)	6 (35,3)	2 (11,8)	17 (100,0)	8 (47,1)	3 (17,6)	4 (23,5)	2 (11,8)	17 (100,0)
Normal	49 (52,1)	45 (47,9)	94 (100,0)	24 (25,5)	25 (26,6)	21 (22,4)	24 (25,5)	94 (100,0)	29 (30,9)	27 (28,7)	18 (19,1)	20 (21,3)	94 (100,0)
Total	67 (54,0)	57 (46,0)	124 (100,0)	34 (27,4)	30 (24,2)	32 (25,8)	28 (22,6)	124 (100,0)	40 (32,3)	34 (27,4)	26 (21,0)	24 (19,4)	124 (100,0)
p-valor	0,745			0,153					0,698				
												
**Comp. Pró-social**													
Anormal	0 (0,0)	1 (100,0)	1 (100,0)	0 (0,0)	0 (0,0)	1 (100,0)	0 (0,0)	1 (100,0)	0 (0,0)	0 (0,0)	1 (100,0)	0 (0,0)	1 (100,0)
Limítrofe	2 (40,0)	3 (60,0)	5 (100,0)	0 (0,0)	4 (80,0)	0 (0,0)	1 (20,0)	5 (100,0)	1 (20,0)	3 (60,0)	0 (0,0)	1 (20,0)	5 (100,0)
Normal	65 (55,1)	53 (44,9)	118 (100,0)	34 (28,8)	26 (22,0)	31 (26,3)	27 (22,9)	118 (100,0)	39 (33,1)	31 (26,3)	25 (21,1)	23 (19,5)	118 (100,0)
Total	67 (54,0)	57 (46,0)	124 (100,0)	34 (27,4)	30 (24,2)	32 (25,8)	28 (22,6)	124 (100,0)	40 (32,3)	34 (27,4)	26 (21,0)	24 (19,4)	124 (100,0)
p-valor	0,444			0,06					0,314				
**Classificação total**													
Anormal	7 (77,8)	2 (22,2)	9 (100,0)	2 (22,2)	3 (33,3)	3 (33,3)	1 (11,2)	9 (100,0)	3 (33,3)	3 (33,3)	2 (22,2)	1 (11,2)	9 (100,0)
Limítrofe	6 (50,0)	6 (50,0)	12 (100,0)	3 (25,0)	3 (25,0)	3 (25,0)	3 (25,0)	12 (100,0)	3 (25,0)	3 (25,0)	3 (25,0)	3 (25,0)	12 (100,0)
Normal	54 (52,4)	49 (47,6)	103 (100,0)	29 (28,2)	24 (23,3)	26 (25,2)	24 (23,3)	103 (100,0)	34 (33,0)	28 (27,2)	21 (20,4)	20 (19,4)	103 (100,0)
Total	67 (54,0)	57 (46,0)	124 (100,0)	34 (27,4)	30 (24,2)	32 (25,8)	28 (22,6)	124 (100,0)	40 (32,3)	34 (27,4)	26 (21,0)	24 (19,4)	124 (100,0)
p-valor	0,328			0,973					0,986				

Teste Qui-quadrado de Pearson

**Legenda:** N = número de participantes; Fem. = Sexo Feminino; Masc. = Sexo Masculino SDQ = *Strengths and Difficulties Questionnaire*; Comp. = Comportamento

Na análise de associação entre as escalas, comportamento pró-social e escore total do questionário SDQ e a Escala de Motivação para aprendizagem, encontrou-se resultados com significância estatística entre “Problemas de Conduta” e “Meta aprender” (p=0,013) com maior média e mediana para o resultado normal, ou seja, a maior proporção de estudantes orientados para a Meta-aprender tiveram resultado normal na subescala de problemas de conduta. Verificou-se ainda associação entre “Problemas de relacionamento com colegas” e “Meta Performance Evitação” (p=0,002) com maior média para o resultado anormal, indicando que esta meta está relacionada, nesta amostra, à maior apresentação de problemas de relacionamento com colegas. Pode-se observar, ainda, associação estatisticamente significativa entre “Classificação total” do SDQ com “Meta Aprender” (p=0,025), com maior média e mediana para o resultado normal, e com “Meta Performance Evitação” (p=0,012), com maior média e mediana para o resultado anormal. Ficou evidente uma relação de proporção entre a preferência das metas e os comportamentos externalizados pelos estudantes (Tabela 3). Pode-se observar, ainda, que a amostra se comportou de maneira bastante coerente na comparação dos testes SDQ e EMAPRE, uma vez que quanto melhor o desempenho na escala de Comportamento Pró-social, maior a preferência para a “Meta Aprender”. Já na análise da “Meta Performance Evitação”, observou-se menor proporção de adolescentes dentro da normalidade no SDQ. Além disso, observou-se pontos de *outliers*, valores discrepantes que fogem do padrão da amostra, da “Meta Performance Evitação” dentro da normalidade*,* o que pressupõe que um comportamento positivo é menos observado nos estudantes com preferência a esta meta de aprendizagem (Figuras 1 e 2).

**Tabela 3:** Análise de associação entre o questionário SDQ e tópicos da Escala de Motivação para Aprendizagem

**Variáveis**	**Meta Aprender**							**Meta Performance Aproximação**						**Meta Performance Evitação**						
	**N**		**Média**		**Mediana**		**D.P.**	**N**	**Média**	**Mediana**		**D.P.**		**N**	**Média**		**Mediana**		**D.P.**	
**Sintomas Emocionais**																				
Anormal	24		28,29		29,50		5,15	24	15,63	15,00		4,65		24	11,63		10,00		4,86	
Limítrofe	16		28,00		29,00		5,23	16	15,19	14,50		5,24		16	9,19		8,00		2,43	
Normal	84		29,81		31,00		4,35	84	15,23	14,50		4,14		84	8,80		8,00		2,19	
p-valor	0,322							0,881						0,777						
**Problemas de Conduta**																				
Anormal	18		26,06		26,00		5,49	18	17,22	16,50		4,72		18	10,44		8,50		4,29	
Limítrofe	16		28,19		28,50		5,13	16	15,31	14,50		4,57		16	9,00		7,50		3,41	
Normal	90		30,12		31,00		4,08	90	14,91	13,50		4,19		90	9,26		9,00		2,74	
p-valor	0,013*							0,168						0,360						
**Hiperatividade**																				
Anormal	18		27,22		27,50		5,50	18	16,83	16,50		4,72		18	11,11		8,50		4,91	
Limítrofe	17		28,12		29,00		3,60	17	14,94	14,00		4,42		17	10,24		9,00		3,90	
Normal	89		29,92		31,00		4,50	89	15,06	15,00		4,26		89	8,89		8,00		2,23	
p-valor	0,059							0,300						0,261						
**Problemas de relacionamento com os colegas**																				
Anormal	13		27,69		28,00		5,19	13	16,54	16,00		5,41		13	12,08		10,00		4,41	
Limítrofe	17		28,94		31,00		6,37	17	15,76	15,00		4,66		17	9,88		10,00		2,40	
Normal	94		29,56		31,00		4,21	94	15,04	14,50		4,16		94	8,94		8,00		2,92	
p-valor	0,470							0,622						0,002*						
**Comp. Pró-social**																				
Anormal	1	21,00		21,00		0,00		1	12,00		12,00		0,00	1		10,00		10,00		0,00
Limítrofe	5	27,00		26,00		2,92		5	8,26		13,00		5,59	5		9,00		7,00		3,46
Normal	118	29,45		31,00		4,65		118	15,33		15,00		4,34	118		9,41		8,00		3,11
p-valor	0,105							0,715						0,585						
**Classificação total**																				
Anormal	9	26,56		28,00		5,55		9	16,33		16,00		5,50	9		14,33		15,00		5,85
Limítrofe	12	26,33		27,00		5,25		12	17,50		16,50		4,64	12		10,08		9,00		3,32
Normal	103	29,86		31,00		4,82		103	14,95		14,00		4,18	103		8,88		8,00		5,22
p-valor	0,025*							0,151						0,012*						

Teste Kruskal-Wallis

* = valor de p≤0,05

**Legenda:** N = número de indivíduos; D.P. = desvio padrão; Comp. = Comportamento

## DISCUSSÃO

O presente estudo demonstrou associação entre os “Problemas de conduta” e “Meta Aprender”, relação entre os “Problemas de relacionamento com os pares” e a “Meta Performance evitação”, bem como da “Meta Aprender” com “Classificação total” do SDQ “normal” e “Meta performance evitação” com “Classificação total” do SDQ “anormal”.

A maior tendência à Meta Aprender demonstrou uma maior orientação à uma motivação intrínseca, que é associada na literatura com um melhor desempenho acadêmico e comportamento com menor indício de ansiedade clínica, sendo reconhecido pelos professores, como alunos que mais aprendem, com maiores índices de autoconhecimento acadêmico e autocontrole em geral^(14)^. Outros estudos apontam que, à medida que o estudante desenvolve maior autoeficácia, possui maior capacidade de gestão emocional, o que otimiza a sua aprendizagem^(15,16)^. Pesquisa recente menciona os hábitos de vida saudáveis como grandes potencializadores da motivação acadêmica^(16)^. Apesar disso, a relação entre motivação e o desempenho acadêmico não é determinante, e isso evidencia que as práticas pedagógicas, apesar de tradicionalmente partirem da motivação extrínseca, possuem uma grande influência sobre a vontade do aluno em cumprir com as tarefas escolares^(14)^.

A maior tendência à Meta Aprender era um resultado esperado, uma vez que é uma amostra sem queixas escolares, o que corrobora a literatura ao mostrar que a motivação mais positiva e duradoura evidencia que questões comportamentais não só podem resultar de uma possível dificuldade escolar, como podem, em muitos casos, preceder às dificuldades escolares^(17)^. Outro fator que deve ser levado em consideração é o observado em um estudo^(8)^ que aponta como o estudante internaliza as crenças dos professores para o seu desempenho acadêmico. Segundo Hareli e Weiner, "todo aluno é sensível às reações das outras pessoas, tanto reais como potenciais, com repercussões sobre seus próprios julgamentos e comportamentos", atestando o fato de que a autopercepção do aluno é também influenciada pelo *feedback* dos professores, e o estudante, por sua vez, responde à esta crença do professor por meio do seu comportamento, seja de raiva, retraimento ou mesmo se expondo mais aos desafios como é demonstrado nas atitudes daqueles que se encaixam na Meta Aprender de maneira majoritária. Isso demonstra que essa tendência é também moldada pelo contexto no qual o estudante está inserido. No contexto da educação superior, um estudo permitiu observar que a motivação é influenciada pela dinâmica ambiental e a forma como o professor conduz as atividades acadêmicas. Isso evidencia que a maior liberdade de apresentar as dúvidas em voz alta, dialogando com o grupo e com o docente, contribuiu para que os estudantes vivessem situações de sucesso frente à aprendizagem, resultando no maior envolvimento nas tarefas^(18)^, pois quando é estimulada a autonomia do estudante, juntamente com outras necessidades psicológicas básicas, ocorre maior desenvolvimento da motivação intrínseca^(19)^.

Apesar de não ter sido verificada associação com significância estatística entre os comportamentos sociais e os dados sociodemográficos, a maior tendência do gênero feminino em apresentar sintomas emocionais e problemas de relacionamento com colegas é compatível com os dados previstos na literatura. Estudos sobre os padrões de comportamento entre os gêneros apontam uma maior frequência de sintomas emocionais, como dor de cabeça, dor de barriga e enjoo, desânimo, crises de choro, perda de confiança, dentre outros, em meninas, enquanto nos meninos há uma maior presença de comportamentos mais agressivos, com maior tendência à hiperatividade^(20,21)^. É necessário maiores investigações deste dado em uma amostra mais diversificada em termos sociodemográficos.

A associação do SDQ com o EMAPRE evidenciou que os adolescentes que apresentaram maior tendência à Meta Aprender apresentaram menos Problemas de Conduta, o que corrobora a literatura^(6)^, que menciona a Meta Aprender como relacionada à motivação intrínseca, que diz respeito a um comportamento positivo. Essa correlação é novamente evidenciada na Classificação Total da associação dos testes SDQ e EMAPRE, em que há uma maior concentração dos adolescentes que optaram pela Meta Aprender no escore comportamental dentro dos padrões de normalidade. Em pesquisa^(22)^ que compara estudantes diagnosticados com Transtorno do Déficit de Atenção e Hiperatividade (TDAH) com crianças que possuem desenvolvimento típico, ficou evidenciada a correlação do transtorno à dificuldade de engajamento nas tarefas acadêmicas, de forma que estes estudantes possuem comportamento compatível com uma motivação de caráter extrínseco.

A coocorrência de alterações comportamentais com a Meta Performance-Evitação é apresentada na associação da Meta Performance Evitação com os problemas de relacionamento com os colegas e a Classificação Total do SDQ. Este resultado é compatível com estudo^(2)^ que observou uma tendência à Meta Performance-Evitação associada ao baixo interesse acadêmico, ansiedade e desempenho escolar mais baixo. Isso também corrobora pesquisa recente^(23)^ realizada em um Centro de Atenção, Aprendizagem e Memória vinculado à Universidade de Cambridge, Reino Unido, com uma amostra mista de alunos com dificuldade escolares, que apresentavam transtornos associados ou não, em que foi realizada associação entre duas escalas SDQ e RCADS-P (Revised Child Anxiety and Depression Scale - RCADS), que avalia a ansiedade e os transtornos depressivos em crianças, e verificou que grande parte da amostra apresentou comportamento anormal no SDQ para a escala de sintomas emocionais (49%) e que os alunos com maiores sintomas emocionais e hiperatividade apontaram para problemas de ansiedade e depressão. Outro estudo^(24)^ que buscou investigar o público infantil evidenciou nas crianças com transtornos de desenvolvimento associados às dificuldades escolares, uma maior presença de comorbidades comportamentais, com a presença de baixa autoestima, maior dificuldade na interação social e comportamento agressivo, se comparado a crianças com diagnóstico isolado. Esses dados ressaltam a importância da correlação de testes, como no presente estudo, que abarquem diferentes fatores envolvidos no processo de aprendizagem.

É possível considerar como limitação do estudo a homogeneidade da amostra com relação aos dados socioeconômicos e aspectos culturais, pois trata-se de estudantes de uma escola de financiamento privado, que não representam a realidade massiva brasileira.

Vale destacar que os avanços alcançados possibilitam compreender as associações entre aspectos comportamentais e motivação para aprender, pois são escassos os estudos com tal triangulação e sobretudo com os protocolos EMAPRE e SDQ, abrangendo a faixa etária aqui apresentada. Desse modo, estas evidências podem contribuir na discussão da temática para profissionais da saúde e educação que lidam com escolares em sua rotina diária.

Como visto, o estudo da temática aponta a integração da aprendizagem com os aspectos emocionais baseado em uma perspectiva multifatorial buscando ressaltar um aspecto subjetivo e de grande importância nos processos de ensino e aprendizagem, a motivação para aprender. Deste modo, o presente estudo assume um papel no diálogo entre os saberes de diversas áreas, possibilita colaborar para a discussão e ampliação do entendimento dos processos relacionados às situações de aprendizagem.

## CONCLUSÃO

A presente pesquisa evidenciou que há associação entre os aspectos comportamentais e a motivação para aprender em adolescentes do Ensino Fundamental por meio da associação entre os escores alterados do SDQ-Por e a Meta Performance Evitação e entre os escores normais do SDQ-Por e a Meta Aprender. Observou-se associação com significância estatística na associação entre o domínio “Problemas de conduta” e a “Meta Aprender”, o domínio “Problemas de relacionamento com os pares” e a “Meta Performance Evitação”, bem como da “Meta Aprender” com “Classificação total” do SDQ “normal” e “Meta Performance Evitação” com “Classificação total” do SDQ “anormal”. A amostra investigada não demonstrou diferença com significância estatística nos aspectos comportamentais e motivação para aprender de acordo com a idade, gênero e ano escolar.

Dessa forma, compreender a relação entre os aspectos comportamentais e a motivação para aprender favorecerá uma abordagem multidisciplinar do adolescente, tanto na prática escolar quanto na clínica fonoaudiológica, com vistas ao bem-estar e a promoção de melhor qualidade de vida da população estudada.
